# UPLC-qToF-MS^E^ Metabolomics to Investigate Century-Old Botanicals: A Case Study of Medicinal Aconite

**DOI:** 10.1021/acsomega.5c11200

**Published:** 2026-08-03

**Authors:** Ella T. Vardeman, Jiyan Ni, Matthew Pace, Edward J. Kennelly

**Affiliations:** † PhD Program in Biology, The Graduate Center, 1371 City University of New York, 365 Fifth Avenue, New York, New York 10016, United States; ‡ Department of Biological Sciences, Lehman College, 2008 City University of New York, 250 Bedford Park Boulevard, Bronx, New York 10468, United States; § The Center for Plants, People and Culture, 2004 New York Botanical Garden, 29000 Southern Boulevard, Bronx, New York 10458, United States; ∥ The William and Lynda Steere Herbarium, New York Botanical Garden, 29000 Southern Boulevard, Bronx, New York 10458, United States

## Abstract

Despite their importance in botanical research, many herbaria worldwide have closed due to high maintenance costs and limited funding. These collections are especially valuable today, in the era of plant metabolomics, as they allow access to species that are typically inaccessible or otherwise difficult to recollect, enabling analysis through both untargeted and supervised methods. This study highlights this potential by analyzing historic samples of medicinal *Aconitum* from the New York Botanical Garden Rusby Collection. We hypothesized that applying modern LC–MS metabolomic techniques to historic plant collections would reveal chemical variation in medicinally important metabolites over time. Untargeted analysis facilitated chemotaxonomic identification of unidentified *Aconitum* samples in the collection. Three diester diterpenoid alkaloids, along with their derivatives, were identified and compared across specimens. Even after more than a century, several of these compounds were detectable, although detection varied among samples. Supervised analysis was used to identify additional chemical features useful as *Aconitum* marker compounds at the species level. By curating and prioritizing historic collections, future researchers can better understand the phytochemical profiles of key medicinal plants and how these profiles change over time.

## Graphical abstract

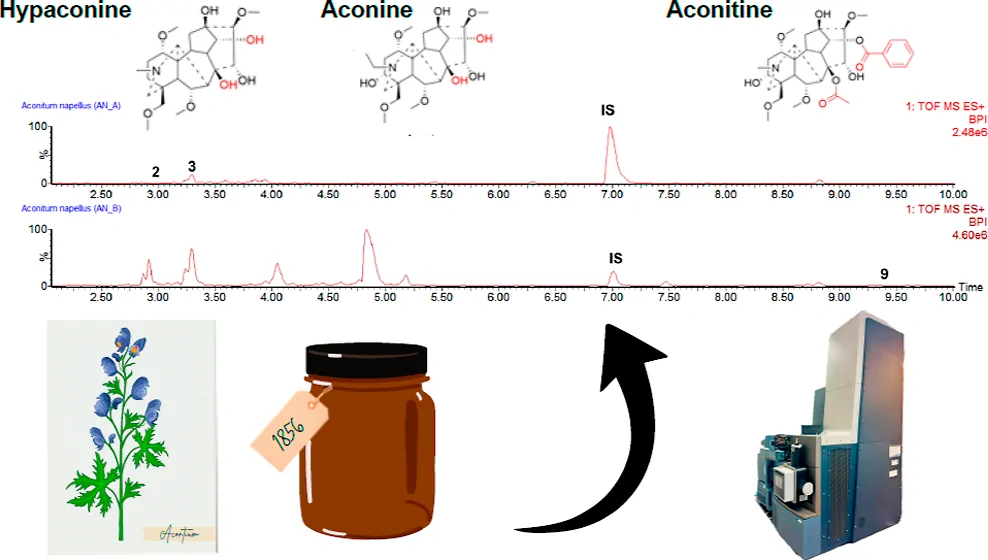

## 1 Introduction

Herbaria, collections of preserved plant specimens and related data, serve as a crucial resource for understanding the history of plant life on Earth, and these collections are becoming increasing important to the study of plant metabolomics.^1^ Over 400 million herbarium specimens are stored in more than 3000 herbaria worldwide, with the number increasing each year as botanists make new collections.^2^ Recent digitization efforts across herbaria have made specimens and their data more accessible to researchers and the public than ever before;^3,4^ however, despite their vital role in research, many herbaria face many pressures, and every year some close due to high maintenance costs, shifting administrative priorities, and limited funding.^5^ While many of these specimens will be relocated to other herbaria, the decision to neglect herbaria, especially historic collections, underscores a lack of awareness of their invaluable contributions to our understanding of the natural world and cultural diversity.^6^ Herbarium specimens are central to documenting new plant species and providing evidence for ongoing systematic revisions and changes to nomenclature.^7^ Beyond morphological comparison, these specimens can be analyzed using various methods, including genomics and metabolomics, to understand the similarities and differences between plants across species.^3^ Furthermore, herbarium specimens can provide materials for research where plant collection would not be feasible by making samples of extinct, endangered, hard to collect, or larger sample sets more widely available.^8^ Recently, a collaborative effort involving 163 herbaria across 48 countries reassessed the phylogeny of flowering plants using genetic sampling of their collections.^9^ This study was unique in scale and included 58% of the currently accepted genera of angiosperms, and 1/3 of the data collected was from herbarium specimens, including historic specimens over 200 years old.

Each herbarium specimen includes dried or preserved plant material and their associated collection metadata such as collection team, collection location, date, habitat information, and/or other associated data such as cultural information. While the included data may be variable depending on the collector, it can inform ecological and conservation studies of plants in a region.^7^ For example, the distribution of non-native and rare plants introduced to a particular region can be assessed by reviewing herbarium collections.^10^ Specimens can also contain biocultural information provided by Indigenous and local communities in ethnobotanical surveys, such as uses, names, and/or management practices.^11^ In the realm of plant metabolomics, metadata describing the plant sample can be useful for applying statistical analyses, like in orthogonal partial least-squares-discriminant analysis (OPLS-DA) analyses.^12,13^ The vast amount of data housed in herbaria, which are now becoming increasingly accessible through digitization efforts, can be used to answer research questions ranging from the effects of climate change on a specific genus to the diversity of medicinal plants used by a culture.^1^

Phytochemical analyses of herbarium specimens, especially historic collections, remain understudied despite being a valuable resource for chemotaxonomy, environmental bioindicators, and drug discovery and authentication.^14^ Historic herbarium collections are often not prioritized in plant genomic and metabolomic studies due to concerns about the stability of small molecules and biopolymers.^8,15^ However, nearly two decades ago, our research group utilized The New York Botanical Garden Rusby Collection to assess the stability of bioactive triterpenoid glycosides in the medicinal plant *Actaea racemosa* L. (black cohosh) over time,^16^ allowing us to sample specimens that were then over 80 years old. Our findings suggested that these chemical compounds not only remained stable over time, but also retained their antioxidant activity. Several other studies have also used metabolomic methods to assess historic collections, however, they are still under represented in the literature.^15,17–19^

Historic herbarium collections offer a unique perspective on plant metabolomics, since some samples are over one hundred years old.^1^ For example, the Rusby Collection at the New York Botanical Garden (NYBG) was collected by botanist and physician Henry Hurd Rusby (1855–1940). In the late 1800s, Rusby traveled to South America to collect bioactive plants used as medicines for Parke, Davis, and Company.^16^ His large collection formed the basis for the NYBG “Economic Museum” and was actively curated from 1899 to the 1940s. The scope and public availability of his collection changed over the decades, and it was put into and taken out storage several times. Most recently, this collection was again made available to the research community through a now completed National Science Foundation award (#2140478). While the Rusby Collection was preserved, many other such collections remain overlooked and forgotten at institutions unaware of their value.^1,16^ This collection and others like it provide a snapshot into the chemistry and use of beneficial plant species, showing how they are affected by factors like stability, diversity, distribution, and change over time.

The genus *Aconitum* L. (Ranunculaceae) includes over 300 species known for their ancient medicinal and toxic properties, which are linked to their diterpenoid alkaloid content.^20,21^ European *Aconitum napellus* L. appears in many Greek and Roman works as a popular poison, although it was also valued for its analgesic and anesthetic properties.^22^ Other *Aconitum* species, such as *Aconitum ferox* Wall. ex Ser., are still widely used in Chinese and Ayurvedic medicine for similar purposes.^23^ Around the time of Rusby’s historic collections of medicinal plants, aconite (*A. napellus*) was included in the 1850 edition of the United States Pharmacopeia.^24^ Samples of *A. napellus*, *Aconitum ferox,* and two unknown *Aconitum* species were part of the historic Rusby collection at NYBG.

We hypothesize that using state-of-the-art metabolomic tools will demonstrate the value of historic collections for natural products research by utilizing the Rusby Collection again, focusing on a different medicinal plant, *Aconitum*. Our aim is to compare the presence of medicinal diterpenoid alkaloids in historic *Aconitum* specimens from the Rusby Collection with those of recently collected *Aconitum*. Our work focuses on the analgesic activity of *Aconitum* diterpenoid alkaloids, such as lappaconitine. The main medicinal component in *Aconitum* is alkaloid aconitine, which was first isolated by Pallus in 1770 (Figure 1).^22,25^ Studies of Paleolithic arrow poisons have shown that aconitine can still be detected on ancient artifacts.^26^ However, other studies suggest that aconitine and related alkaloids are not stable in different storage conditions.^27^ We further hypothesize that medicinal diterpenoid alkaloids remain in historic *Aconitum* specimens, though at lower concentrations than in new samples. Understanding how bioactive compounds degrade over time can be helpful information for medicinal plants like *Aconitum*, which are still widely used around the world. Metabolomic methods for analyzing these compounds in historic collections can also be useful for chemical characterization and chemotaxonomy of medicinal plant species.

**Figure 1:**
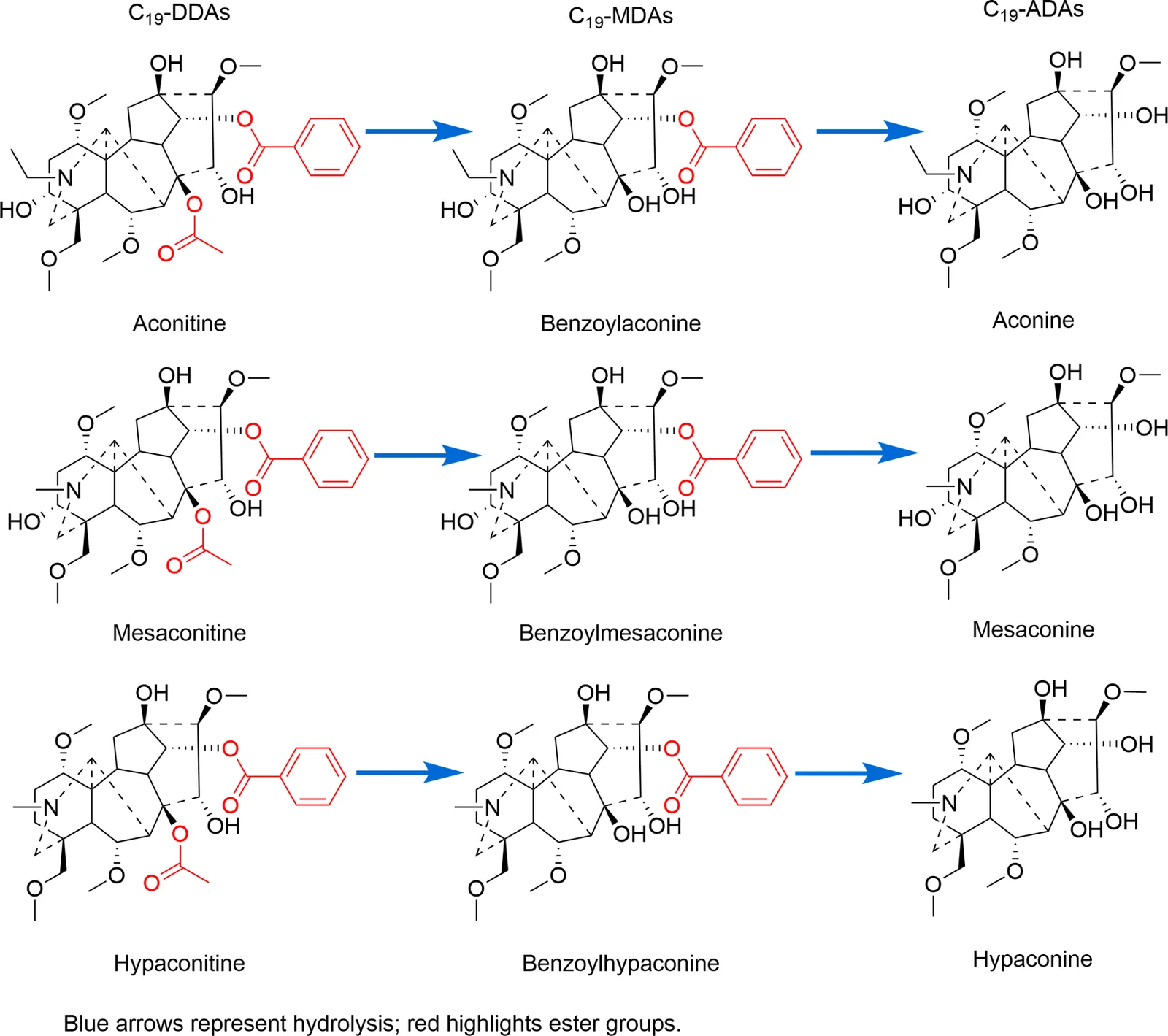
Diterpenoid alkaloids found in *Aconitum* spp.

## 2 Materials and Methods

### 2.1 Sample Collection

Fresh *A. napellus* L. (Digging Dog Nursery; Albion, CA) were grown in the Lehman College greenhouses. All samples were deposited in the NY Herbarium. *Aconitum* samples from the Rusby Collection (*Aconitum ferox* Wall ex Ser., *A. napellus*, and unknown *Aconitum* spp.) in the NY Herbarium were also analyzed (Table 1); samples are digitized and available online through the C.V. Starr Virtual Herbarium (https://sweetgum.nybg.org/science/vh/). Samples were prepared for metabolomic analysis by grinding roots into a powder, extracting with 80% methanol, sonicated for 20 min, and filtered using Whatman (Maidstone, United Kingdom) Uniflo 0.45 μm syringe filters. All samples were prepared at a concentration of 2 mg/mL in LC–MS grade 80% methanol containing 10 μg/mL palmatine (Shyuanye Biotechnical Company, China; 98% purity) as an internal standard. Identification of *Aconitum* diterpenoid alkaloids (aconitine, mesaconitine, hypaconitine, benzoylaconine, benzoylmesaconine, and benzolyhypaconine) was confirmed by coinjection with chemical standards purchased from Shyuanye Biotechnical Company.

**Table 1:** Metadata on *Aconitum* Samples Analyzed

sample ID	species	voucher number	collection date	collection site
AF-A	*Aconitum ferox*	collector unknown 313 (Barcode 200038)*	unknown	Darjeeling, Punjab, India
AF-B	*Aconitum ferox*	collector unknown 312 (Barcode 200050)*	unknown	Darjeeling, Punjab, India
AN-A	*Aconitum napellus*	H. H. Rusby 1864*	unknown	unknown
AN-B	*Aconitum napellus*	H. H. Rusby 318*	unknown	Hazara, Punjab, India
AS-A	*Aconitum* sp.	collector unknown 1906 (Barcode 719331)*	unknown	Japan
AS-B	*Aconitum* sp.	H. H. Rusby 1856*	unknown	unknown
AN-1	*Aconitum napellus*	Ni 100	2025	Lehman College Greenhouses, Bronx, NY (Digging Dog Nursery)
AN-1	*Aconitum napellus*	Ni 101	2025	Lehman College Greenhouses, Bronx, NY (Digging Dog Nursery)
AN-3	*Aconitum napellus*	Ni 102	2025	Lehman College Greenhouses, Bronx, NY (Digging Dog Nursery)

### 2.2 UPLC-qToF-MS Analysis

*Aconitum* samples were analyzed using electrospray positive mode (ESI^+^) with an ACQUITY UPLC coupled to a Xevo G2 QToF-MS (Waters, Milford, MA). The analysis employed an ACQUITY UPLC BEH C18 Column, 1.7 μm, 2.1 × 50 mm with ACQUITY UPLC BEH C18 Column, 1.7 μm, 2.1 × 5 mm precolumn kept at 40 °C. The solvent gradient involved MS-grade water with 0.1% formic acid (solvent A) and MS-grade acetonitrile with 0.1% formic acid (solvent B). The flow rate was set at 0.5 mL/min, following this gradient: 0–10 min, 100–60% A; 10–12 min, 40–100% B; 12–13 min, 100% B; 14–15 min, 0–100% A. Triplicate analyses of the extract, blanks, and QC samples (containing equal concentrations of tested extracts) were performed using 1 μL injections with needle overfill. Lock mass correction employed leucine-enkephalin. Key settings included: 3 kV capillary voltage, 3 V sampling cone, 30 V extraction cone, 100 °C source temperature, and 450 °C desolvation temperature. Nitrogen desolvation flow was 800 L/h. Data acquisition used MS^e^ mode over 100–1500 Da with a scan time of 0.2 s. LC–MS data were processed with Progenesis QI and EZInfo (Waters Corporation, Milford, MA) for compound identification and quantification. Compound abundances were normalized to the internal standard palmatine (6.94_352.1555 *m*/*z*). An in-house chemical library for *Aconitum* spp. was compiled using SciFinder-n (American Chemical Society, https://scifinder-n.cas.org/) for compound identification in Progenesis QI.^13^

## 3 Results and Discussion

### 3.1 Untargeted Metabolomic Analysis

Untargeted metabolomic methods can be used to compare phytochemical differences across a group of plant samples. For example, untargeted methods can be applied to compare chemical variation across different tissues in the same plant species.^13^ These methods can also be used to aid in understanding the chemotaxonomy of unidentified plant samples.^28,29^ Principal component analysis (PCA) is a type of untargeted method that reduces high-dimensional data, such as LC–MS data, into fewer dimensions, facilitating the visualization of trends and patterns.^30^ PCA analysis divided *Aconitum* samples into three groups: (1) historic *A. napellus*; (2) historic *Aconitum ferox* and historic unknown *Aconitum* samples; (3) newly collected *A. napellus* samples (Figure 2). Other studies have established chemical differences across *Aconitum* species.^31,32^ These results indicate that the unidentified samples are likely *Aconitum ferox* samples as well, thus demonstrating the potential of using metabolomic methods for chemotaxonomy of historic collections.

**Figure 2:**
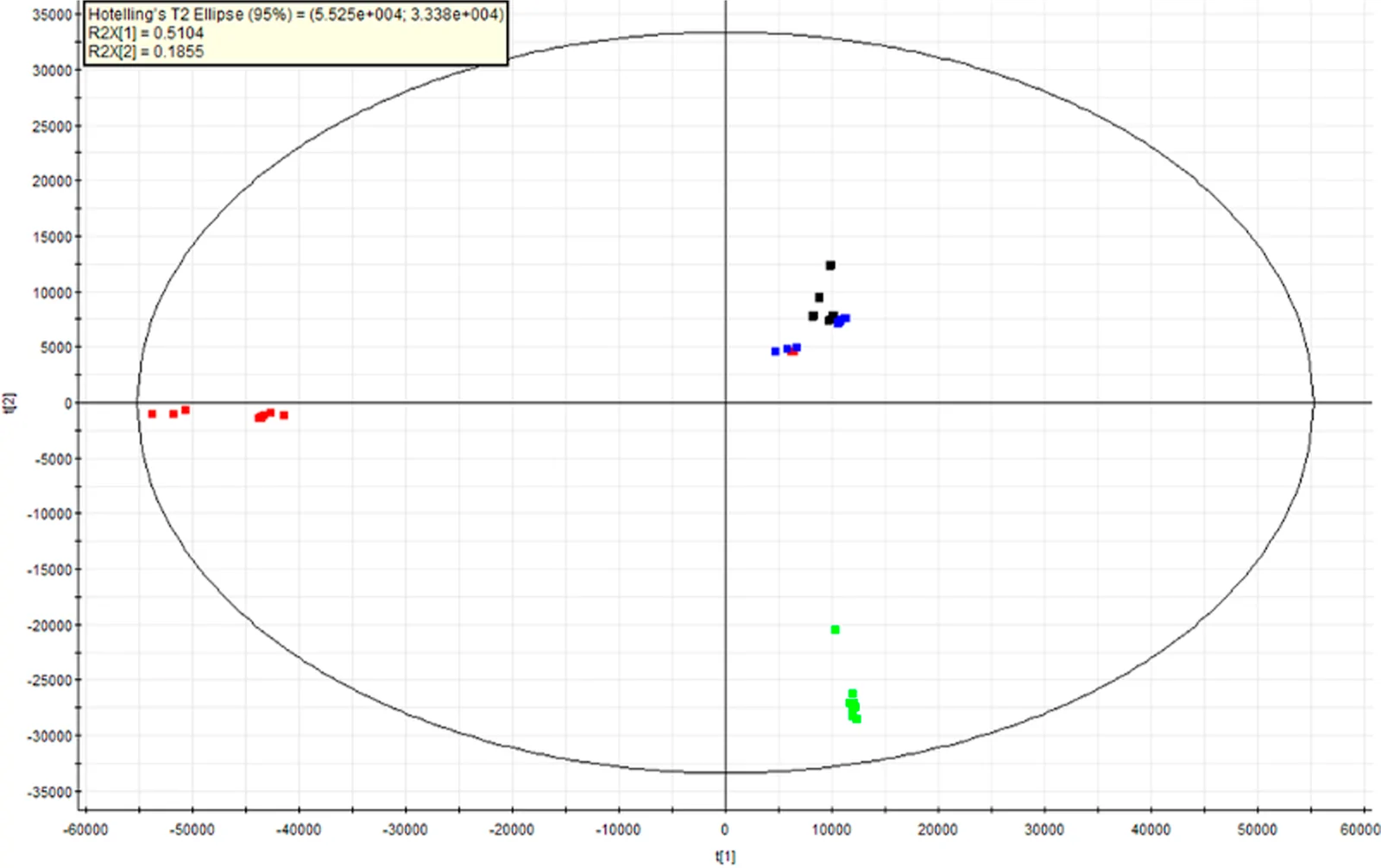
Principal component analysis (PCA) of historic *Aconitum ferox* (black), historic *Aconitum napellus* (red), historic unidentified *Aconitum* (blue), and newly collected *Aconitum napeullus* (green).

### 3.2 Identification of Prominent *Aconitum* Alkaloids

Aconitine and its derivatives mesaconitine and hypaconitine are the primary bioactive diterpenoid alkaloids found in *Aconitum* species, and are the focus of most phytochemical investigations of *Aconitum* species.^33^ These compounds are diester diterpenoid alkaloids (DDAs) and are highly toxic. In traditional Chinese medicine, various methods are employed to process and reduce the toxicity of *Aconitum* by hydrolyzing toxic DDAs into less toxic monoester diterpenoid alkaloids (MDAs) (Figure 1). In general, a salt solution is typically applied to expedite hydrolysis of the DDAs. The roots are then heated and introduced to water, either through boiling or steaming.^25,34^ This allows for the hydrolysis of DDAs into MDAs bezoylaconine, benzoylmesaconine, and benzoylhypaconitine. These compounds can be hydrolyzed a second time to form aminoalcohol diterpenoid alkaloids (ADAs), aconine, mesaconine, and hypaconine.^35^ This hydrolysis can also occur naturally over time as herbarium specimens deteriorate, especially depending on the storage conditions.^14,27^ Although these diterpenoid alkaloids are known to be unstable and degrade over time, other studies focusing on historic *Aconitum* have used aconitine as a marker compound.^25,26^

Aconitine, mesaconitine, hypaconitine, and their six hydrolyzed derivatives were identified in both historic and freshly collected *Aconitum* samples using ProGenesis QI (Figure 3; Table 2). However, comparison of LC–MS chromatograms across historic samples found that the presence of these compounds was inconsistent across samples. For example, samples AF_B (historic *Aconitum ferox*) and AS_A (historic unidentified *Aconitum*) contained the most complete profiles of the nine diterpenoid alkaloids, while similar samples such as AF_A (historic *Aconitum ferox*) and AS_B (historic unidentified *Aconitum*) contained little or none of the same compounds (Figure 4). Aconitine (9) was the most abundant compound found in newly collected *A. napellus* samples, but was found in only half of the historic samples at low levels (Figure 5). Overall, hydrolyzed derivatives of aconitine, mesaconitine, and hypaconine (1–6) were present more frequently in historic collections. This is likely due to the degradation of aconitine, mescaconitine, and hypaconitine since the time of collection.^14,27^ Aconine (3) was the only derivative identified in newly collected samples. Aconine can be a useful marker compound for *Aconitum* samples.

**Figure 3:**
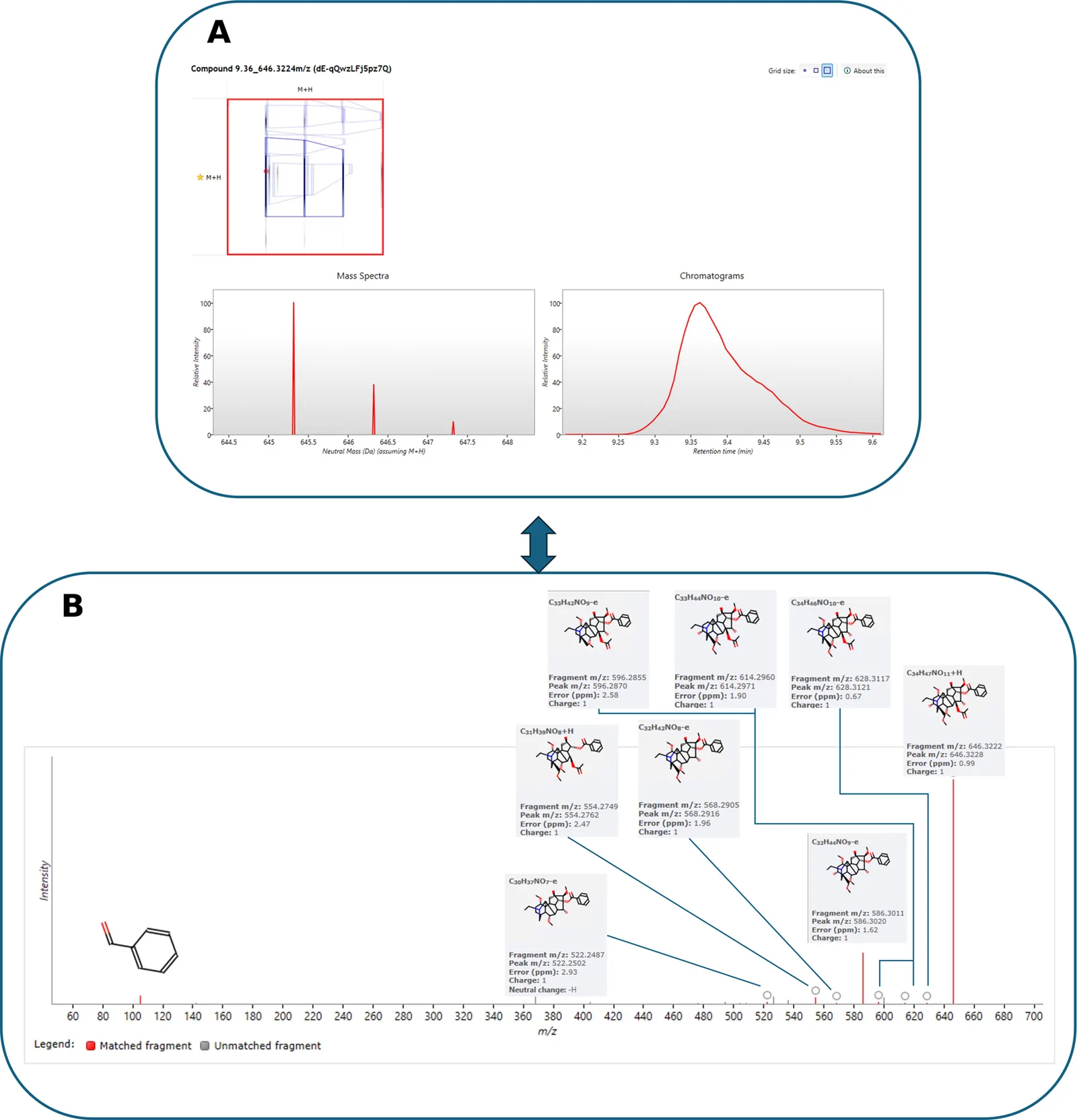
Progenesis QI showing an identification of aconitine from the filtered metabolic features (A) Identification of adducts common for aconitine; (B) identification of aconitine. The experimental spectra which are matched against the fragment ions are highlighted in red. The circle on the top of the red spectra shows the structure, measured *m*/*z*, theoretical *m*/*z*, and corresponding mass error in ppm.

**Table 2:** Identification of Well-Known Bioactive Alkaloids in *Aconitum* Samples^a^

compound number	retention time (RT, min)	[M + H]^+^ (molecular formula (MF), ppm)	identification	fragmental ion exact mass [M – X]^+^ (MF, ppm)
1	2.81	486.2701 [M + H]^+^ (C_24_H_40_NO_9_, 0.71)	mesaconine	
2	2.93	470.2748 [M + H]^+^ (C_24_H_40_NO_8_, −0.02)	hypaconine	
3	3.22	500.23858 [M + H]^+^ (C_25_H_40_NO_9_, 0.69)	aconine	
4	6.76	590.2965 [M + H]^+^ (C_31_H_44_NO_10_, 0.95)	benzoylmesaconine*	540.2592 [M + H – H_2_O – CH_3_OH]^+^ (C_30_H_38_NO_8_, 1.93);
				105.0335 [M + H – C_24_H_39_NO_9_]^+^ (C_7_H_5_O, 11.58)
5	7.14	604.3122 [M + H]^+^ (C_32_H_46_NO_10_, 0.98)	benzoylaconine*	554.2749 [M + H – H_2_O – CH_3_OH ]^+^ (C_31_H_40_NO_8_, 2.40);
				105.0335 [M + H – C_25_H_41_NO_9_ ]^+^ (C_7_H_5_O, 10.86)
6	7.56	574.3014 [M + H]^+^ (C_31_H_44_NO_9_, 0.57)	benzolyhypaconine*	
7	8.68	632.3068 [M + H]^+^ (C_33_H_45_NO_11_, 0.38)	mesaconitine*	572.2855 [M + H – CH_3_COOH]^+^ (C_31_H_42_NO_9_, 1.62);
				105.0335 [M + H – C_26_H_40_NO_10_ ]^+^ (C_7_H_5_O, 10.49)
8	9.23	616.3117 [M + H]^+^ (C_33_H_46_NO_10_, 0.09)	hypaconitine*	556.2905 [M + H – CH_3_COOH]^+^ (C_31_H_42_NO_8_, 1.45)
9	9.36	646.3224 [M + H]^+^ (C_34_H_48_NO_11_, 0.34)	aconitine*	586.3011 [M + H – CH_3_COOH]^+^ (C_32_H_44_NO_9_, 1.62);
				105.0335 [M + H – C_27_H_43_NO_10_ ]^+^ (C_7_H_5_O, 11.73)

a Mean abundance compared between historic and newly collected *Aconitum napellus* (AN).* denotes chemical features validated by co-injection.

**Figure 4:**
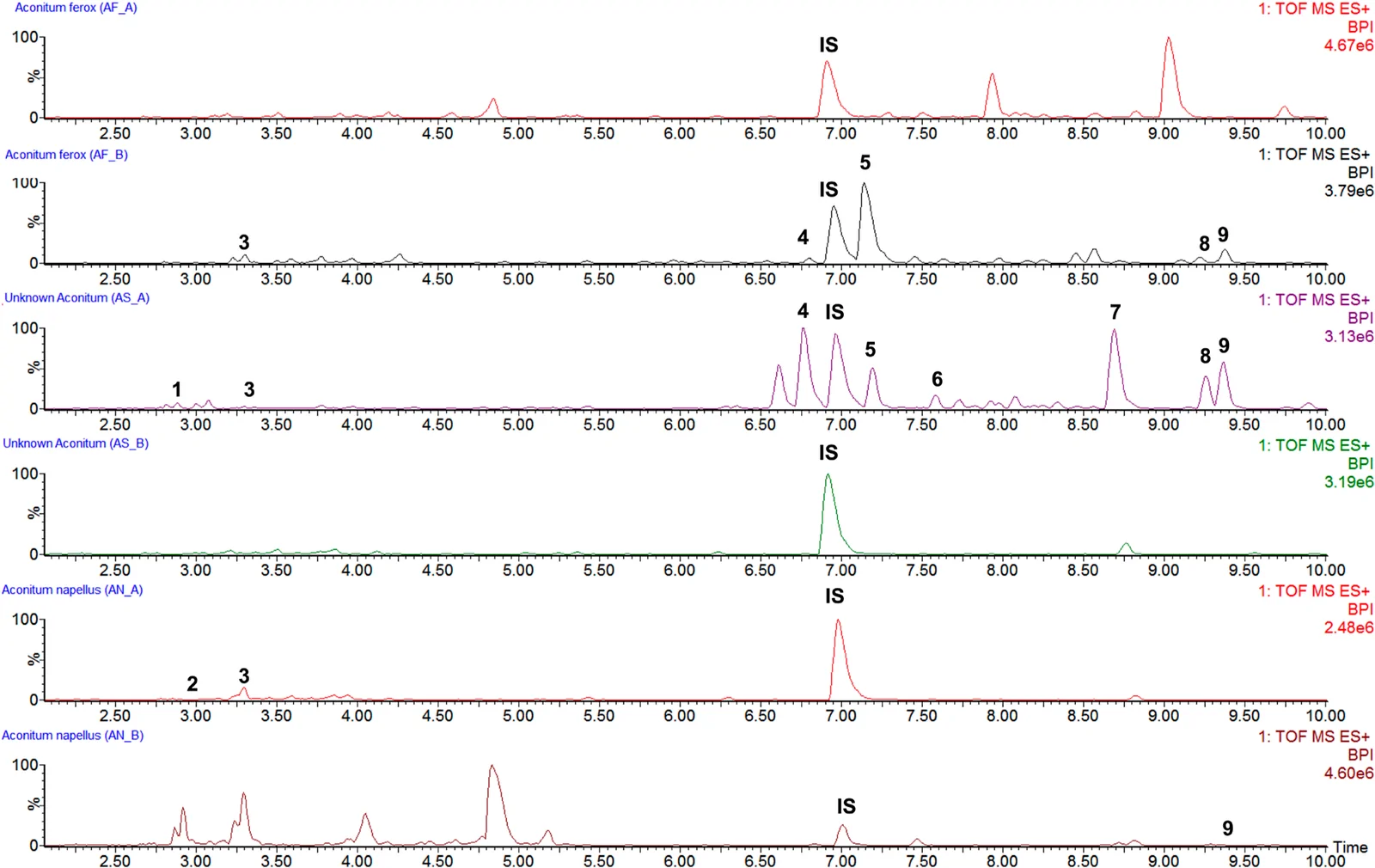
LC–MS base peak intensity (BPI) chromatograms denoting *Aconitum* alkaloids (1–6) in historic specimens listed from top to bottom AF_A, AF_B, AS_A, AS_B, AN_A, and AN_B and the internal standard, palmatine (IS).

**Figure 5:**
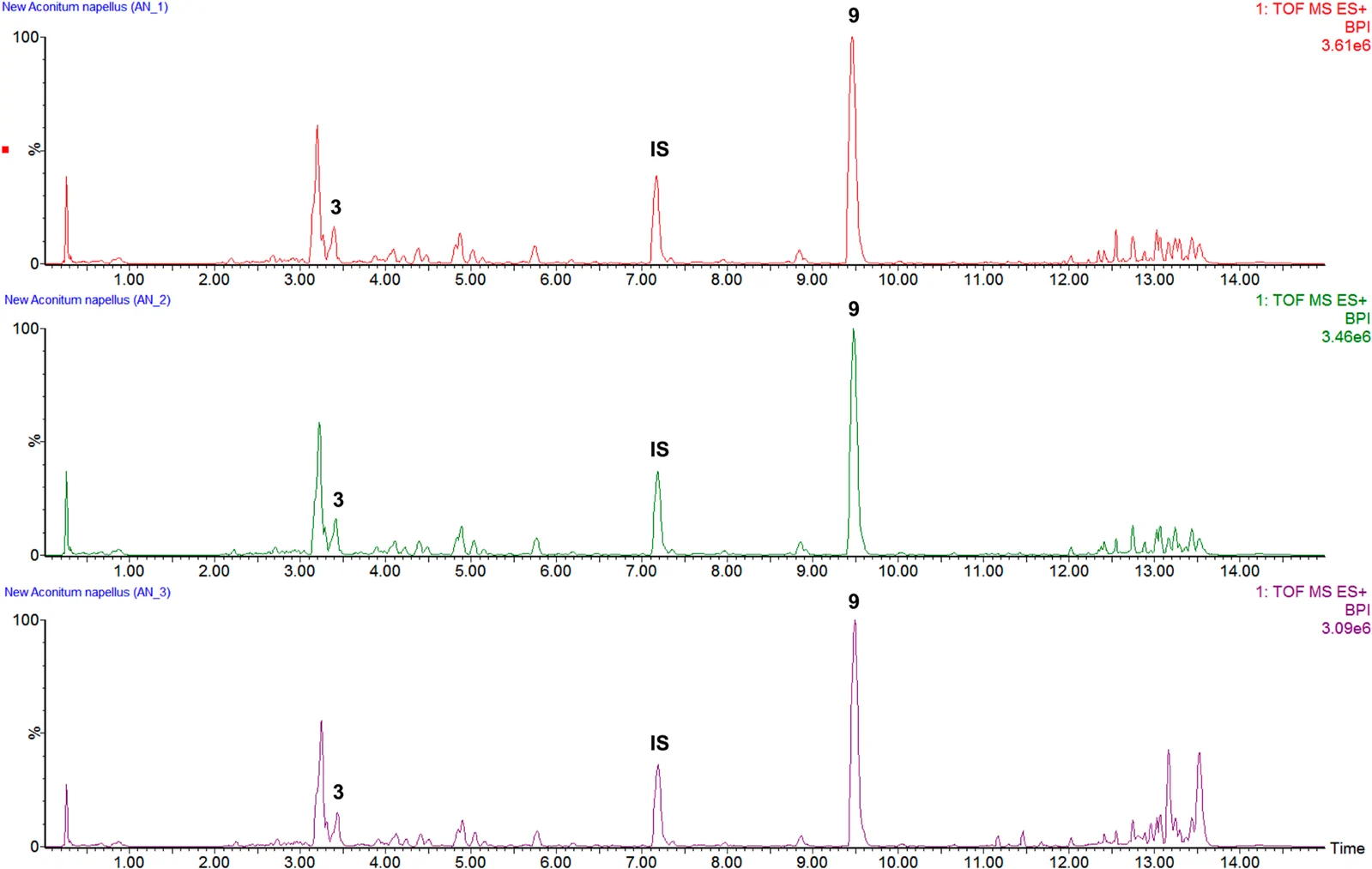
LC–MS BPI chromatograms denoting *Aconitum* alkaloids (1–9) in newly collected *Aconitum napellus* specimens from top to bottom AN_1, AN_2, and AN_3 and the internal standard, palamatine (IS).

Despite the instability of aconitine and related alkaloids, they can still be found in samples over 100 years old. However, their presence was highly variable and likely related to how individual specimens were stored. Storage conditions for the Rusby samples are unknown, especially in the years following the closure of the Rusby Collection in the 1940s, however it is unlikely they were housed under modern archival conditions, and were likely subject to high and fluctuating temperatures and humidity. It is unlikely that the herbarium curators would have imagined the useful chemical features that would be retained in these samples a century later. Regardless, this exemplifies the importance of maintaining historic and new herbarium collections as these samples are as important to scientific discovery the day they were collected as they are decades or even centuries later. Future studies continuing to investigate the degradation of important medicinal compounds in samples over time can provide a better understanding of popular medicinals, both past and present.

### 3.3 Supervised OPLS-DA Analysis and Identification of Additional *Aconitum* Marker Compounds

Since the nine target diterpenoid alkaloids were inconsistently present across historic samples, we aimed to identify additional marker compounds beyond aconitine and its derivatives in these samples that could be useful to others studying historic *Aconitum* specimens. While untargeted methods may allude to broad chemical differences between plant samples, supervised analysis builds a model to determine significant chemical features between two groups.^36^ For example, untargeted methods, like OPLS-DA, can be combined with bioactivity data to hypothesize marker compounds associated with active plant samples.^12^ For this study, OPLS-DA was used to create a model to compare chemical features between historic and newly collected *A. napellus* samples, thus prioritizing features preserved in historic samples for identification with Progenesis QI. Features with *p*[1] < −0.5 were targeted for identification (Figure 6), resulting in 33 chemical features associated with historic *A. napellus* samples (Table S1). Several of these were identified as other alkaloids known to occur in *Aconitum* species, including casamine. Hypaconine (2) was the only aconitine derivative identified in this model. The relative abundance of these compounds was compared across historic *Aconitum ferox*, historic unidentified *Aconitum*, and newly collected *A. napellus* samples (Figure 7). No single compound was present in all three groups, primarily due to chemical differences between *Aconitum ferox* and *A. napellus*, rather than between historic and modern samples. Focusing on chemical features shared between historic and new *A. napellus* could be valuable for future research, specifically on *A. napellus*.

**Figure 6:**
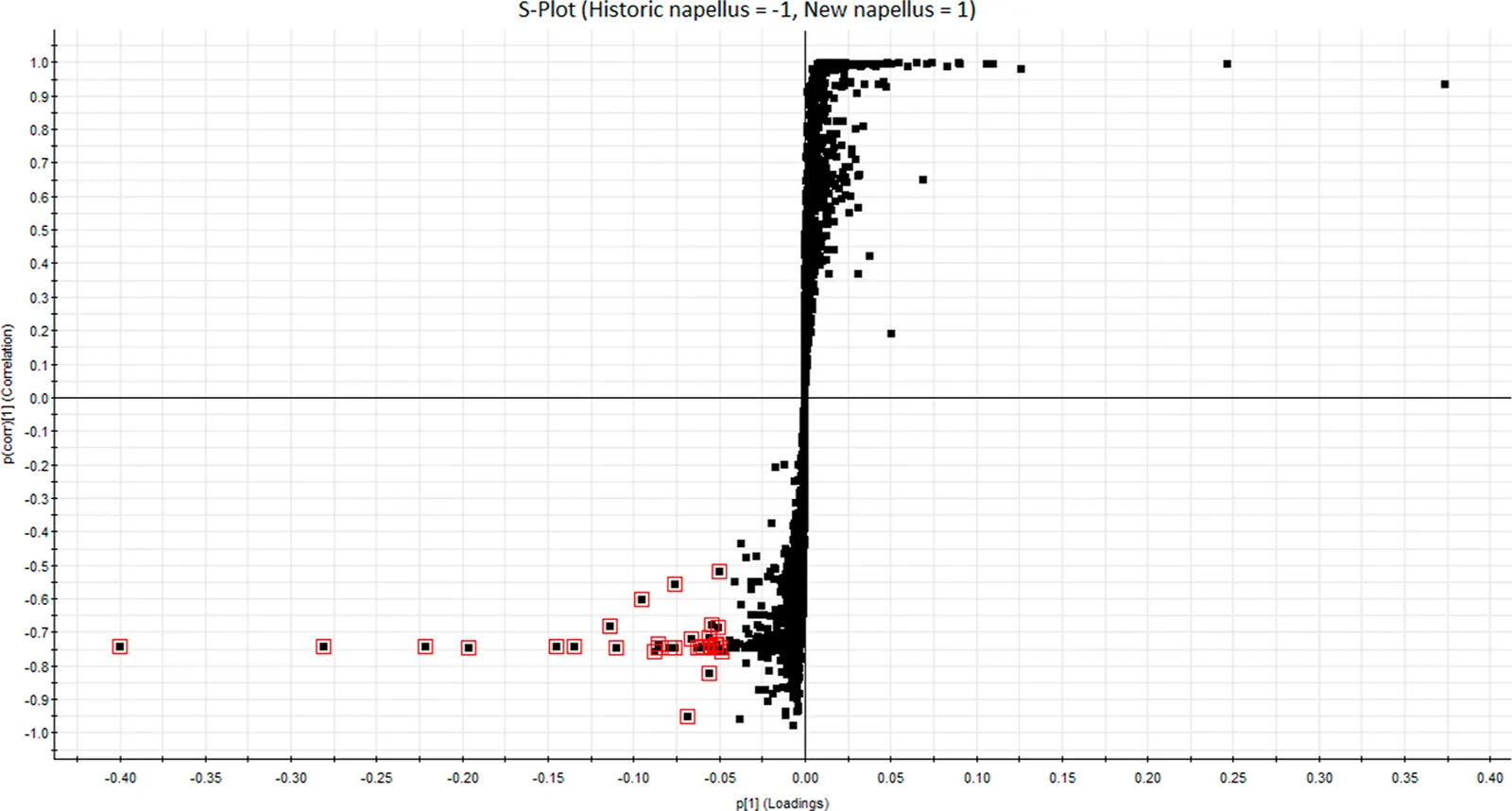
S-plot identifying significant chemical features associated with historic samples (*p* ≤ −0.05) from OPLS-DA analysis of historic and newly collected *Aconitum napellus.* R2Y (cum) = 0.993; Q2 (cum) = 0.978.

**Figure 7:**
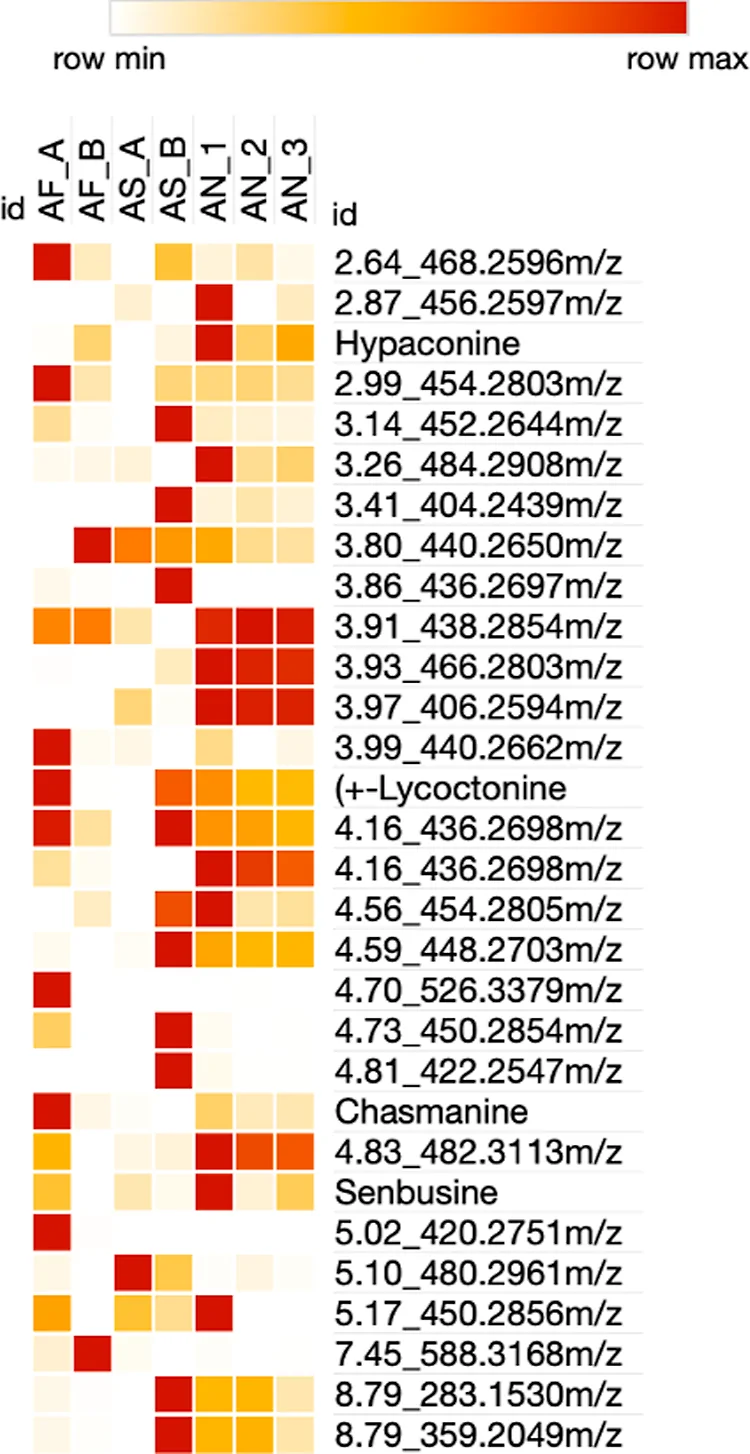
Heat map depicting the relative abundance of marker compounds, prioritized by OPLS-DA (Figure 4), detected in historic *Acontitum ferox* (AF_A, AF_B), historic unknown samples (AS_A, AS_B), and newly collected *Aconitum napellus* (AN_1, AN_2, AN_3). Created with Morpheus.

## 4 Conclusion

This study highlights the importance of analyzing historic collections for plant metabolomics research. Aconitine and its derivatives are the primary focus of phytochemical literature on *Aconitum* species. While these compounds are known to degrade, we were still able to detect several compounds in samples over a century old that were not maintained in archival conditions over the past 100 years. By curating and prioritizing historic collections, future studies can gain a more comprehensive understanding of the phytochemical profiles of key medicinal plants and how they endure and evolve over time. Moreover, unidentified samples like those in this study can be chemotaxonomically identified with tools like untargeted metabolomics.

## Supplementary Material



## Notes

Table of tentatively identified marker compounds for historic *A. napellus* samples identified by OPLS-DA model (PDF)

## ⊥

School of Medicine, Emory University, Atlanta, Georgia, United States.

## Abbreviations

BPI: base peak intensity
NYBG: New York Botanical Garden
OPLS-DA: orthogonal partial least-squares-discriminant analysis
PCA: principal component analysis
QC: quality control
