# Supplementary material for: UPLC-qToF-MSE Metabolomics to Investigate Century-Old Botanicals: A Case Study of Medicinal Aconite
Source: ACS Omega. 2026 Aug 3;11(32):47276–84. doi: 10.1021/acsomega.5c11200 (PMC13491871; doi:10.1021/acsomega.5c11200)
Supplement: Supplementary file 1 [file ao5c11200_si_001.pdf]

UPLC-qToF- MS^E Metabolomics to Investigate Century-old Botanicals: A Case Study of Medicinal Aconite

Ella T. Vardeman[1,2,3†], Jiyan Ni[1,2], Matthew Pace[4], and Edward J. Kennelly[1,2*]

AUTHOR ADDRESSES

[1]PhD Program in Biology, The Graduate Center, City University of New York, 365 Fifth Avenue, New York 10016, USA

[2]Department of Biological Sciences, Lehman College, City University of New York, 250 Bedford Park Boulevard, Bronx, New York 10468

[3]The Center for Plants, People and Culture, New York Botanical Garden, 29000 Southern Boulevard, Bronx, NY 10458

[4]The William and Lynda Steere Herbarium, New York Botanical Garden, 29000 Southern Boulevard, Bronx, NY 10458

Footnotes

† Current affiliation: School of Medicine, Emory University, Atlanta, Georgia, United States

Table S1. Tentatively identified marker co mpounds for historic *Aconitum napellus* samples identified by the OPLS-DA model.

RT_EM	Identification	Molecular formula	Adducts	ppm	p[1]	p(corr)[1]
2.64_468.2596 *m/z*	Unidentified				-0.0519	-0.746086
2.87_456.2597 *m/z*	Unidentified				-0.196	-0.746371
2.93_470.2748 *m/z*	Hypaconine	$C_{24}H_{39}NO_8$	[M+H]	-0.02	-0.0856	-0.747591
2.99_454.2803 *m/z*	Unidentified				-0.0754	-0.746916
3.14_452.2644 *m/z*	Unidentified				-0.088	-0.758285
3.26_484.2908 *m/z*	Unidentified				-0.2812	-0.74254
3.41_404.2439 *m/z*	Unidentified				-0.0554	-0.823055
3.80_440.2650 *m/z*	Unidentified				-0.0553	-0.749249
3.86_436.2697 *m/z*	Unidentified				-0.0683	-0.950972
3.91_438.2854 *m/z*	Unidentified				-0.1134	-0.683489
3.93_466.2803 *m/z*	Unidentified				-0.0761	-0.559613
3.97_406.2594 *m/z*	Unidentified				-0.0518	-0.739603
3.99_440.2662 *m/z*	Unidentified				-0.0607	-0.748692

4.03_468.2960 *m/z*	(+)-Lycoctonine	$C_{25}H_{41}NO_7$	[M+H]	0.84	-0.2212	-0.745453
4.03_484.2552 *m/z*	Unidentified				-0.0496	-0.747899
4.16_436.2698 *m/z*	Unidentified				-0.0789	-0.748611
4.38_422.2905 *m/z*	Unidentified				-0.0554	-0.718037
4.56_454.2805 *m/z*	Unidentified				-0.0552	-0.745672
4.59_448.2703 *m/z*	Unidentified				-0.0853	-0.736649
4.70_526.3379 *m/z*	Unidentified				-0.0621	-0.749463
4.73_450.2854 *m/z*	Unidentified				-0.1341	-0.745849
4.81_422.2547 *m/z*	Unidentified				-0.0553	-0.749557
4.83_452.3011 *m/z*	Chasmanine	$C_{25}H_{41}NO_6$	[M+H]	1	-0.3996	-0.744779
4.83_482.3113 *m/z*	Unidentified				-0.0663	-0.721413
4.84_452.3723 *m/z*	Unidentified				-0.0492	-0.749196
4.94_424.2700 *m/z*	Senbusine A	$C_{23}H_{37}NO_6$	[M+H]	1.6	-0.11	-0.746379
5.02_420.2751 *m/z*	Unidentified				-0.0591	-0.74294
5.10_480.2961 *m/z*	Unidentified				-0.0515	-0.73714
5.17_450.2856 *m/z*	Unidentified				-0.1452	-0.745812

7.45_588.3168 *m/z*	Unidentified				-0.0953	-0.604773
8.71_570.3066 *m/z*	Unidentified				-0.0495	-0.518457
8.79_283.1530 *m/z*	Unidentified				-0.0503	-0.688773
8.79_359.2049 *m/z*	Unidentified				-0.0545	-0.681161
